# Macrophages in heterotopic ossification: from mechanisms to therapy

**DOI:** 10.1038/s41536-021-00178-4

**Published:** 2021-10-26

**Authors:** Yifei Huang, Xinyi Wang, Daixuan Zhou, Wenwen Zhou, Fengyi Dai, Hui Lin

**Affiliations:** 1 First Clinical Medical School, Nanchang University, 330006 Nanchang, Jiangxi Province China; 2 Queen Marry university, Nanchang University, 330006 Nanchang, Jiangxi Province China; 3 Second Clinical Medical School, Nanchang University, 330006 Nanchang, Jiangxi Province China; 4 Department of Pathophysiology, School of Basic Medical Sciences, Nanchang University, 330006 Nanchang, Jiangxi Province China; 5 Department of Surgery, Brigham and Women’s Hospital, Boston, MA 02115 USA

**Keywords:** Immunological disorders, Trauma

## Abstract

Heterotopic ossification (HO) is the formation of extraskeletal bone in non-osseous tissues. It is caused by an injury that stimulates abnormal tissue healing and regeneration, and inflammation is involved in this process. It is worth noting that macrophages are crucial mediators of inflammation. In this regard, abundant macrophages are recruited to the HO site and contribute to HO progression. Macrophages can acquire different functional phenotypes and promote mesenchymal stem cell (MSC) osteogenic differentiation, chondrogenic differentiation, and angiogenesis by expressing cytokines and other factors such as the transforming growth factor-β1 (TGF-β1), bone morphogenetic protein (BMP), activin A (Act A), oncostatin M (OSM), substance P (SP), neurotrophin-3 (NT-3), and vascular endothelial growth factor (VEGF). In addition, macrophages significantly contribute to the hypoxic microenvironment, which primarily drives HO progression. Thus, these have led to an interest in the role of macrophages in HO by exploring whether HO is a “butterfly effect” event. Heterogeneous macrophages are regarded as the “butterflies” that drive a sequence of events and ultimately promote HO. In this review, we discuss how the recruitment of macrophages contributes to HO progression. In particular, we review the molecular mechanisms through which macrophages participate in MSC osteogenic differentiation, angiogenesis, and the hypoxic microenvironment. Understanding the diverse role of macrophages may unveil potential targets for the prevention and treatment of HO.

## Introduction

Heterotopic ossification (HO) is the formation of extraskeletal bone in non-osseous tissues caused by abnormal differentiation of progenitor cells, because of local or systemic pathological imbalances^1^. In general, HO is classified into two types^2^: acquired HO (AHO) and genetic HO (GHO). Inflammation contributes to the onset and progression of these HO types^3^. Studies show that macrophages, as critical mediators of the immune system^4^, are interesting players that link inflammation and HO pathogenesis^5^. In fact, increasing research evidence chronicles a close relationship between macrophages and HO, showing that the recruitment and activation of macrophages drive HO^6–8^. It is the activation of macrophages that regulates the critical processes of HO development, including osteogenic differentiation and chondrogenic differentiation of mesenchymal stem cells (MSCs)^8^, a suitable hypoxic microenvironment^9^, and angiogenesis^10^. In this review, we discuss the important role of macrophages in HO and highlight macrophage-centered therapeutic strategies, which effectively modulate inflammation and macrophages, including immunotherapy and nanomedicine. Gaining insights into these issues will shed light on the critical role of macrophages in HO pathogenesis and lead to novel therapeutic strategies for HO.

## Heterotopic ossification

**Fig. 1:**
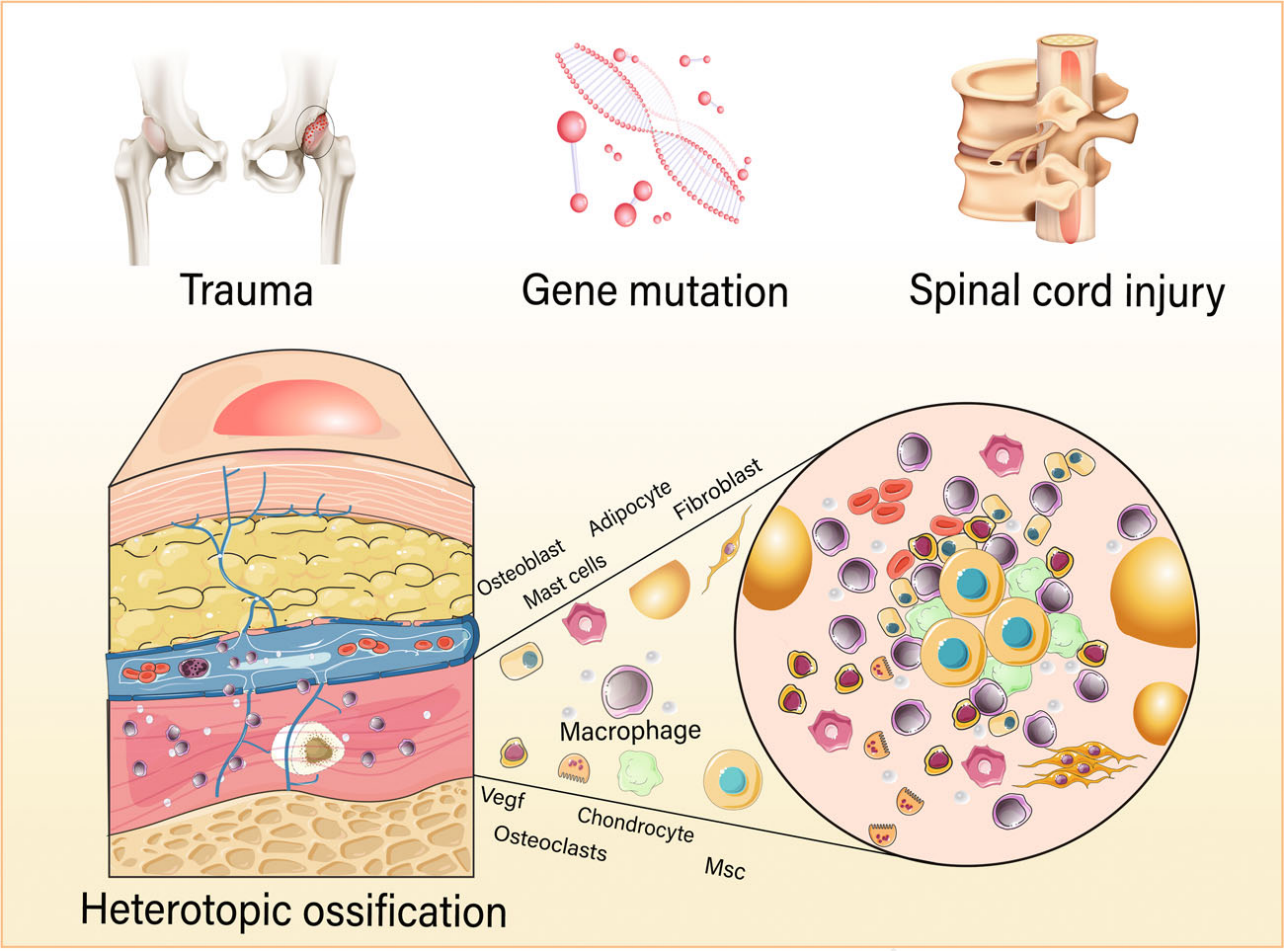
Schematic illustration of heterotopic ossification (HO) formation. HO is the formation of extraskeletal bone in soft tissues, which is caused by neurogenic trauma (spinal cord injury, encephalitis, *etc.*), gene mutation, and severe skeletal muscle trauma. HO is characterized as a multifactorial pathology and inflammatory disease. Inflammation is a common feature of acquired and genetic HO, which manifests as pain, warmth, redness, and swelling. Macrophages, mast cells, MSCs, chondrocytes, bone cells, fibroblasts, osteoclasts, and osteoblasts exist in lesion areas of vessels and muscles. Macrophages in the inflammatory environment influence osteogenic differentiation of MSCs and angiogenesis, the key steps of HO development.

HO is largely thought to be caused by injuries that stimulate abnormal tissue healing and regeneration mediated by inflammation^11^. AHO, a non-genetic form, is the most common and constitutes a severe complication of musculoskeletal trauma, including severe burns, fractures, joint arthroplasty (traumatic HO, THO)^12^, and cerebral or spinal insult (neurogenic HO, NHO)^13^ (Fig. 1). The pathogenesis of AHO is complex and remains unclear. Studies^14,15^ have shown that local inflammation and physical damage cause AHO. These lead to the recruitment of progenitor cells and release of numerous osteogenesis-inducing factors, which, accompanied by fibrosis and angiogenesis, eventually induce cartilage intermediates to form ectopic bone^14,15^. Fibrodysplasia ossificans progressiva (FOP) and progressive osseous heteroplasia (POH) are two autosomal dominant genetic forms of HO (GHO)^16^. Patients with FOP carry a mildly activating mutation of *ACVR1*, a gene which encodes the cell surface type I bone morphogenetic protein (BMP) receptor also named ALK2 (activin receptor-like kinase 2)^17^. To date, several mutations have been identified, all of which located in the glycine-serine (GS) region of *ACVR1*. The most common mutation in FOP patients is a single-point mutation of *ACVR1* R206H^17^. This mutation decreases the stability of the glycine-serine region, which leads to the continuous activation of ACVR1, and ultimately HO and joint fusion in FOP patients^18^. Other rare missense mutations in the GS or protein kinase (PK) domain of ACVR1 (G365D, c.617 G > A, c.605 G > T, c.983 G > A) have been reported in FOP patients^19–22^. Genetically POH is caused by loss of function mutations in the Gs-α isoform of the *GNAS1* gene^23^. Gene mutations in FOP and POH induce abnormal homeostasis and cell differentiation processes, triggering ectopic intrachondral or intramembranous ossification^24^. It should be noted that the pathogenesis and progression of AHO are influenced by injury and accompanied by a robust inflammation^25^. On the other hand, the two rare FOP and POH autosomal dominant genetic HO forms have different presentations and clinical severities^16^. The patients with FOP frequently present with redness, swelling, heat, and pain similar to local inflammation before the onset of the disease^26^, and tissue injury enhances the aggravation of FOP development. However, POH is not usually relevant to trauma or inflammation^27^. Studies have reported that the connective tissues, including blood vessels and skeletal muscles at AHO and FOP lesion sites have elevated levels of macrophages, mast cells, MSCs, osteocytes, chondrocytes, fibroblasts, and cytokines^8,28–30^.

In the HO mouse model, co-depletion of macrophages and mast cells effectively blocks the occurrence and development of FOP^8^. Several studies have investigated the role of mast cells in FOP and NHO^14,31^. For example, mast cells participate in the formation of FOP lesions. Factors released by degranulated mast cells stimulate inflammatory edema, fibrosis, and angiogenesis and enhance the progression of FOP and NHO lesions^14,31^. Moreover, in the AHO mouse models, macrophage depletion alone has been reported to significantly impair HO^6^. Studies propose that inflammation is a major risk factor in the development of HO^1,3,32^, and macrophages play a major role in this process^6–8,33^.

## Inflammation and macrophages

Inflammation is a normal physiological response of the body to infections and injuries^34^, but it is also often associated with the occurrence and deterioration of HO^35,36^. Inflammation is a process of damage and repair tightly regulated by the immune and vascular systems, which can be classified into two kinds, namely acute and chronic^34^. In non-onset FOP patients, studies have reported a pro-inflammatory state^36^. This implies that FOP is caused by prolonged and hyper-activation of the immune system mediated by chronic inflammation^36^, whereas AHO occurrence and development are caused by acute or chronic inflammation^37,38^. Inflammation is an important trigger factor for FOP and AHO. Inflammation induces osteogenic gene expression, angiogenesis, and hypoxic microenvironment by stimulating immune cells to secrete numerous inflammatory factors, thereby jointly promoting cartilage differentiation and bone formation in tissues^35,39,40^. In FOP, only minor local inflammation is sufficient to trigger HO^26^. In particular, the effect of activin A (Act A) in an inflammatory environment is unique to FOP, as the binding of Act A to ACVR1 mutants described previously drives HO development in FOP^8,41^. AHO is caused by inflammation with injury, and HO caused by trauma most often occurs as a result of extensive soft tissue injury and inflammation^42^. While it is difficult to attribute HO pathogenesis and progression to a specific type of inflammation^21^, it is vital to point out that macrophages, as the most critical sentinels and regulators of the immune system to inflammation^43^, actually play central roles in this inflammatory disease^8,44^. Macrophages serve as immune cells, as well as active secretory cells that secrete diverse mediators, which regulate the host defense system, inflammation, and homeostasis^45^. Tissue-resident macrophages are derived from the embryonic or adult hematopoietic stem cell (HSC) progenitor cells under homeostatic condition^46^. The study has shown that monocyte-derived macrophages originating from the bone marrow cells are mostly associated with responses to tissue repair and inflammation^47^. Monocyte-macrophage lineage cells are characterized by considerable diversity and plasticity, which not only play a crucial role in regulating homeostasis and promote normal tissue development, including bone morphology repair^48^, fiber formation, branch recovery^49^, and regulation of angiogenesis^50^. However, when these functions are not coordinated, macrophages can also become the root cause of many bone metabolic diseases, such as HO^51^ and rheumatoid arthritis^52^. Although tissue-resident macrophages are evenly distributed in various tissues, mononuclear-macrophages change with time and are highly heterogeneous in vivo^53^, in response to numerous signals. At the most basic level, the M1 macrophages are characterized by high levels of pro-inflammatory cytokine expression and promote inflammation and the killing of microorganisms^54^. M2 macrophages are believed to be involved in the promotion of tissue remodeling and tumor progression^55^. Macrophages in the human body do not exist in the form of a single phenotype. They will dynamically change during injury, regeneration, and healing. M1 macrophages commonly initiate inflammatory response immediately after injury, then during the repair inflammation stage, infiltrating macrophages have an M2 phenotype^56,57^. A study has highlighted the heterogeneity that presents in the macrophages at the site of extremity injury during HO, including different types of macrophages being recruited to the site of HO^5^. Depletion of macrophages is shown to greatly suppress the development of AHO and FOP in some studies^6,8^, but to promotes HO in another study^58^. Although many depleted macrophages were M2, these studies^6,8^ have failed to assess which of the two the macrophages, M1 or M2, is depleted first and more severely. Notably, depletion of M1 may greatly weaken the phagocytic function^51,59^. Therefore, these may indicate that different types of macrophages play roles along with different stages of HO, but not those macrophages must have an inhibitory effect on HO. The appropriate phenotype of macrophages exhausted at a precise time could be key in the prevention and treatment of HO^51^. Because of such a sophisticated and complex system of macrophage polarization, the mechanism by which macrophages affect HO is elusive.

## Molecular mechanism of macrophages in HO

Enrichment of macrophages has been reported in damaged tissue in AHO and FOP, their clearance effectively inhibits HO development, and both M1 and M2 macrophages promote ectopic bone formation in different ways^5^. The three factors necessary for the occurrence and development of HO include an inciting inflammatory incident, osteogenic and chondrogenic differentiation of MSCs, and an appropriate microenvironment^60,61^. Various studies have reported that macrophages contribute critically in modulating these multiple aspects of HO progression from initiation to MSC chondrogenic and osteogenic differentiation, hypoxia microenvironment, and angiogenesis.

## Macrophages regulate osteogenic differentiation of MSCs

**Fig. 2:**
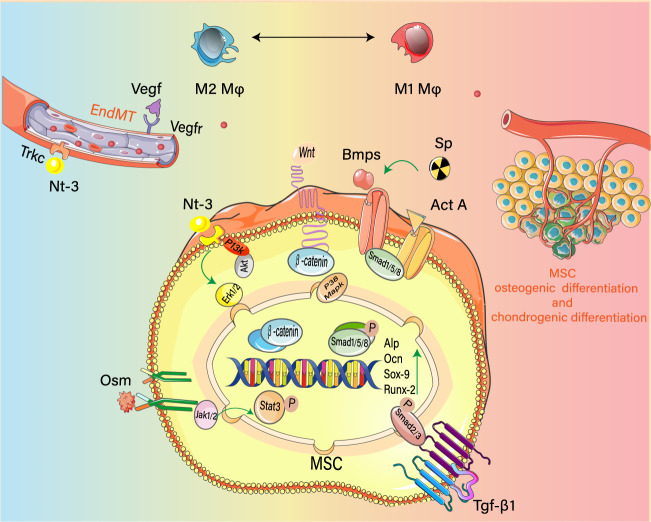
Molecular mechanisms of HO induced by different phenotypes macrophages. Mononuclear macrophages are polarized to the M1-M2 macrophages in injured tissues in responding to injury. Macrophages secrete OSM and TGF-β1, BMPs, Act A, SP, and VEGF, and promote the expression of NT-3 and other factors. These signal molecules through different signal pathways, including TGF-β1-Smad2/3 and BMP-Smad1/5/8, jointly induce osteogenesis of MSCs, angiogenesis, and proper microenvironment formation, promoting the occurrence and development of HO through multiple pathways.

Ectopic bones are formed in soft tissues after severe injury, inflammation, or genetic diseases. Except for POH which is a type of intramembranous ossification, AHO and FOP are associated with endochondral ossification^62^. This usually occurs through initial cartilage formation followed by endochondral ossification. Ectopic bone formation includes four stages: inflammation, cartilage formation, osteogenesis, and ectopic bone maturation. In the inflammatory phase of AHO and FOP, immune cells infiltrate the injury site^63^. In the chondrogenesis stage, MSCs differentiate into chondrocytes^64^. During the osteogenesis stage, chondrocytes undergo hypertrophy and calcification^65^. In the final stage of maturation, the bone marrow matures to form a cancellous bone. Therefore, inflammation is an important factor that initiates ectopic bone formation. Inflammation has different roles during osteogenic differentiation and chondrogenic differentiation. For example, several inflammatory factors such as TGF-β promote cartilage and bone formation^39^. A study on patients with osteoarthritis reports that M1 macrophages induce cartilage apoptosis and then M2 macrophages promote cartilage hypertrophy and ectopic bone formation^66,67^. However, the effect of different types of macrophages on chondrogenic differentiation in HO patients remains unclear. MSCs are multipotent stem cells with self-renewal ability and can differentiate into chondrocytes or osteoblasts^68^. Osteogenic and chondrogenic differentiation of MSCs is one of the key processes responsible for the occurrence and development of HO^60^. It has been chronicled those macrophages of different phenotypes promote the osteogenic differentiation of MSCs^69^, and the exhaustion of macrophages evidently impairs the osteogenic differentiation potential of MSCs^70^. In return, the MSCs release hormone such as prostanoid prostaglandin E-2 (PGE-2) to regulate macrophage polarization^71^. MSCs also regulate macrophage chemotaxis by secreting chemokines such as MIP-1 and 2 and MCP-5, which are chemoattractants for monocytes/macrophages and play a key role in macrophage infiltration during wound healing^72^. Different types of macrophages modulate inflammation and accelerate bone repair growth and HO progression^7,73^. Therefore, the interaction between MSCs and macrophages promotes the development of HO. Currently, it is believed that macrophages secrete cytokines and factors, including TGF-β1, BMP, Act A, OSM, SP, and NT-3. They play a vital role in mediating MSCs osteogenesis differentiation and are associated with both acquired and inherited HO^5,7,8,74–76^ (Fig. 2).

## TGF-β1

TGF-βs constitute three major isoforms (TGF-β1, TGF-β2, and TGF-β3), all of which participate in the pathogenesis and progression of HO formation^5^. However, macrophages only secrete TGF-β1^5^, a key regulator of chondrogenic differentiation and monocyte/macrophage function^77,78^. In the early stages of inflammation, TGF-β1 has been found to be co-localized with F4/80+ and CD68 + macrophages in THO sites of mice and human THO samples, and TGF-β1 is upregulated in many macrophage clusters at HO sites^5^. The high levels of TGF-β1 induce osteogenic differentiation of MSCs through the TGF-β1-Smad2/3 pathway, promoting HO occurrence and progression^5^. Matrine inhibits TGF-β/smad2/3 pathway and induces MSC migration and osteogenic differentiation, and significantly suppresses HO progression in an ATP mouse model^79^ (Fig. 2). The study has also found that the CD47-activating peptide that blocks TGF-β1 for systemic therapy inhibits the formation of HO by altering the monocyte/macrophage phenotype and reducing the levels of chondrogenic and osteogenic markers^5^. However, further studies are needed to understand how phenotypes of macrophages are altered and to determine whether the body produces other heterogeneous macrophages other than M1/M2. The formation of AHO is prevented in macrophage-deficient colony-stimulating factor 1 (CSF-1) deficient (Csf1−/−) mice, which are depleted in macrophages^39^. In addition, HO is not formed in TGF-β1−/− mice whose macrophages/monocyte lineages and neutrophils do not produce TGF-β1, while signs of HO formation in control mice is noted^39^. Therefore, TGF-β1 secreted by immune cells in the inflammatory phase is an important inducer of HO. However, the problem of the study^39^ is that the effect of neutrophils on TGF-β1 is not ruled out, and it is unclear whether macrophages also secrete other cytokines to regulate the process of HO. Hence, at this time, cannot fully confirm that TGF-β1 secreted by macrophages participates in the occurrence and development of AHO. Similar findings are made in hereditary HO^36^. A previous study has reported thatM2 macrophages secretes TGF-β1 during the inflammatory phase in FOP^36^, indicating that TGF-β1 also contributes to the fibrosis and ossification process of FOP.

## BMPs

BMPs are multifunctional growth factors that are involved in bone development. Among them, BMP-2, BMP-4, and BMP-7 are primarily involved in the occurrence of HO^39,80,81^. It is documented that BMPs participate in HO development by promoting the proliferation and differentiation of MSCs into cartilage and bone tissue through the BMP/Smad and p38 MAPK signaling pathways^36,80^. BMPs facilitate the assembly of BMP type I and type II receptors to activate downstream Smad effector proteins 1, 5, and 8. After being phosphorylated by type II receptors, such as BMPRII and ACTRIIA, activated BMP type I receptors, including ALK2, ALK3, and ALK6, can in turn phosphorylate SMAD proteins to induce FOP^82^ and AHO^83^. Another study has found that the interaction between BMPs and the Wnt/β-catenin signaling pathway related to epithelial-mesenchymal transition (EMT) that mediate HO^84^. In addition, modulation of Smad1/5/8 phosphorylation, via small molecule inhibitors targeting BMP type I receptor kinase activity, could mitigate traumatic HO formation resulting from Achilles tenotomy and severe body burn^83^. Knocking out BMP type I receptors (ALK2 and ALK3), as well as using BMP ligand trap A3Fc, have significantly reduced HO formation following Achilles tenotomy^85^. Taken together, these losses of function studies indicate that BMP signaling is critical in traumatic HO and that BMPs represent the most likely candidate injury‐induced osteogenic factor(s) in THO^85^. Trauma is not enough to induce HO, and other factors, such as gene mutation^86^ and neurogenic injury^87^, are needed. For example, the induction of NHO requires either a TBI or a spinal cord injury with muscle damage^88,89^. Blast injury alone cannot cause THO^90^, but when combined with concomitant fracture^91^ or wound infection, such as bacterial infection, it can induce severe HO^92^. On the other hand, hereditary HO, such as FOP, usually only requires gene mutation and mild inflammation, including injury-mediated inflammation and noninjury-mediated inflammation, such as viral infection^26^. BMPs are strongly correlated with the participation of macrophages in inflammation (Fig. 2). Studies have found that the microenvironment recruited by macrophages might well promote the expression of BMP in MSCs and even express BMP, thereby inducing HO^15,44,80^. Histological analysis^80^ confirms the expression of BMP-7 in BMP-2–induced AHO mice muscle tissue at 48 h after injury, but not in control uninjured muscle tissue. It also reveals severe inflammatory cell infiltration into the muscle tissue, indicating the possibility that BMP-7 is a result of the interplay between inflammation and activation of BMP-2. Double immunofluorescence staining indeed confirm the macrophage marker, CD68, to be localized in close proximity to BMP-7 signals^80^. In the AHO mouse model, following the decrease in macrophages, BMP-7 level in injury muscle tissue are also greatly reduced at the early stage^80^. Although these studies have not profiled the macrophage phenotypes that secrets BMPs, both M1 and M2 macrophages could secrete BMP^93,94^ and indirectly enhance the expression of BMPs. In animal studies, TGF-β1 secreted by macrophages has been reported to promote the expression of BMPs in MSCs, inducing ectopic bone formation^95^. Furthermore, the effect of TGF-β1 on BMP is macrophage-dependent^95^. Importantly, the BMP signaling pathway coordinates the polarization of macrophages and subsequently affects inflammation^8^. The secretion of BMP-2 at the remodeling site, which can potentially stimulate M2 commitment, may induce an anti-inflammatory response by polarized macrophages^96^. Furthermore, BMP-4 has been implicated in promoting the secretion of pro-inflammatory factors, *e.g*., TNF-α, by mast cells to activate inflammation in an FOP mouse model^8^. Therefore, the etiology of FOP is considered as a frequent spontaneous inflammation and an abnormal repair program^8^. The interaction between macrophages and BMPs promotes the recruitment and polarization of macrophages that express high levels of BMPs, which mediate the dysregulation of osteogenic differentiation of MSCs and prompting HO occurrence and progression^15^.

Act A, a member of the Activin family, is secreted by immune cells, such as Th2 cells^97^, neutrophils^98^, and natural killer (NK) cells^99^. Although no studies have detected the release of Act A from macrophages^53^, Act A is involved in regulating the polarization of macrophages by inducing the expression of arginase-1 required for the M2 phenotype and inhibiting the expression of the IFN-γ-inducing NO synthase required for the M1 phenotype^97^. As a crucial mediator of inflammation, immunity, and fibrosis^100^, Act A is potentially associated with the occurrence and development of FOP^41^. Act A cannot induce ectopic bone formation in wild-type mice^41^. In contrast, Act A can activate ACVR1 (R206H) to contribute to HO^41^. Hatsellet et al.^41^ has shown that a monoclonal antibody against Act A antibody prevents the occurrence of FOP. Activation of the ACT A alone is not enough to induce the formation of ectopic bones in skeletal muscle, as injury or other types of tissue damage is also required^63^. In an inflammatory environment, by amplifying mutated ACVR1 signaling, Act A induces differentiation of MSCs into osteoblasts and chondrocytes by activating the Smad1/5/8 and mTOR signaling pathways^101^ (Fig. 2), while inhibiting osteoclast formation^102^ causing the FOP form of HO. Moreover, Act A regulates immune cells (including monocytes, macrophages, microglia, mast cells, NK cells, and dendritic cells) and the functions of B cells and T cells. It exhibits pro-inflammatory and anti-inflammatory multifunctional effectors^103^. Experimental evidence^8^ has shown that after introducing the ACVR1 R206H mutation to enhance Act A signaling in the FOP model, the number of mononuclear—macrophages and mast cells increases at the early and middle stages. However, only the mast cells secrete more cytokines, while no difference in cytokine secretion in macrophages^8^ is found. Although the effect of Act A on macrophages and HO is not fully understood, we speculate that the complex interaction among Act A, macrophages, and ACVR1 mutations is responsible for the development of FOP. As Act A only activates the signaling from mutant ACVR1, but not wild-type ACVR1^104^, Act A does not induce the formation of post THO^104^.

## OSM

OSM^105^, a member of the IL-6 cytokine family, is secreted by monocytes, macrophages, and bone cells, and promotes the development of HO^76^ (Fig. 2). Some studies have shown that macrophage-derived OSM plays a key role in NHO^76^ and that elevated levels of macrophage-derived OSMs mediate NHO by activating osteogenic differentiation of MSCs^76^.This is due to the high expression of OSM mRNA in the damaged muscles of NHO mice and patients, and the drastic increase in OSM expression after stimulating monocyte/macrophage with LPS^76^. Treatment with anti-OSM antibody can greatly reduce the mineralization of NHO-MSC induced by osteogenic differentiation medium supplemented with LPS-activated monocytes/macrophages. In vivo and in vitro studies have shown that the binding of the activated macrophage-derived OSM to OSMR indeed promotes the osteogenic and chondrogenic differentiation of MSCs in muscle tissue, thereby enhancing NHO^76^. Deleting the OSMR gene dramatically reduces the chance of NHO development compared with wild-type controls following spinal cord injury and muscle injury in vivo^76^, confirming that signaling through the OSMR promotes HO formation. A subsequent study^74^ has proposed that macrophage-derived OSMs mediate NHO via the binding of OSM to the GP130/OSMR complex on MSCs and then tyrosine-phosphorylation of STAT3 by activating Janus kinase1/2 (JAK1/2)^74^. The signaling pathways synergistically promote osteogenic differentiation of MSCs^74^. STAT3 is significantly higher and remains persistently activated in injured muscles of mice with central nervous system lesion and developing NHO, but not in injured muscles without spinal cord injury^74^. The result shows that this persistent STAT3 phosphorylation and high activation in the injured muscle is an important driver of NHO^74^. The long-lasting activation of STAT3 may require the stimulation of spinal cord injury. The use of a specific inhibitor of JAK1/2 kinases greatly inhibits the progress of NHO in the mice, which supports that the OSM—JAK/STAT3 pathway indeed induces the occurrence and development of HO^74^. However, limitations of this study are that phosphorylation of STAT3 cannot be confirmed in muscle satellite cells or mesenchymal cells in vivo, and that the phenotype of macrophages was not investigated. Furthermore, activation of STAT3 in MSCs upregulates the expression of OSMR and LIFR, which enhances the effect of OSM on MSCs, and improves the osteogenic efficiency of MSCs^106^. Of note, deletion of the OSMR gene has not completely ablated NHO^76^, indicating that in addition to OSM, other cytokines or mechanisms involving in macrophages are also at play.

## SP

SP, a neuropeptide produced by macrophages, is involved in transmitting information during noxious stimulus response, such as pain^107^. In recent years, studies have shown that SP facilitates the proliferation and mineralization of MSCs and inflammation, which are closely linked to AHO and FOP^30^. They also emphasize that damage induces the upregulation of SP, which promotes HO via BMP^14,30^ (Fig. 2). This has been illustrated by the accumulation of high levels of M1 macrophages and SP at the HO site in THO rat models with Achilles tendon injury^75^. Macrophages appear to be involved in the infiltration of substance P^108^, which may stimulate M1 macrophages to secrete high levels of inflammatory factors, resulting in overexpression of BMP-2^75^. CGRP counteracted the effect of SP has been shown to suppress ectopic bone formation effectively^75^. Therefore, SP secreted by M1 macrophages after injury amplifies inflammation and may have an effect on upregulating BMP-2 to mediate THO^75^. BMP-2, in turn, induces the expression of the neuroinflammatory mediators, SP, and CGRP^14^. The role of SP in FOP and NHO has received significant attention. High levels of SP are secreted in the early lesions of NHO, and non-neurogenic SPs are mainly produced by macrophages and mast cells^6^, which significantly promote the osteogenic differentiation of MSCs by upregulating BMP-4^30^.

## NT-3

NT-3 plays an essential role in the growth and development of the nervous system and bones^109^. In the THO mouse models^110^, NT-3 activated by TGF-β cytokines acts on TrkC to induce endothelial to mesenchymal transition (EndMT), leading to MSCs generation. NT-3 accelerates the differentiation of MSCs into bone and cartilage by upregulating Sox9, RUNX2, and other^110^. NT-3 is also able to stimulate neovascularization to promote HO^110^. While inhibition of NT‐3 suppressed the induction of EndMT and bone formation in HO^110^. A recent study indicates that activated macrophages mediate the secretion of NT-3 from chondrocytes that play a vital role in HO formation^7^. Interestingly, NT-3 is always co-localized with M1 and M2 macrophages throughout the formation of HO^7^ (Fig. 2). An in vitro study verified that macrophages mediate the secretion of NT-3^7^. In addition, macrophage-derived NT-3 accelerates osteogenic differentiation of tendon stem cells (TDSC) of mesenchymal lineages by activating the ERK1/2 and PI3K/Akt signaling pathways^7^. Moreover, NT-3 enhances the expression of BMP (especially BMP-2) and VEGF in mineralized cells to mediate bone and blood vessel formation^111^.

TGF-β1, BMPs, Act A, OSM, SP, and NT-3 upregulated by different macrophage phenotypes are involved in the occurrence of HO by regulation of the osteogenic differentiation of MSCs. The soft tissues of HO patients have been shown to harbor elevated levels of inflammatory factors, including MCP-1, IGF-1, and IL-23^112,113^, that can induce MSC osteogenic differentiation via different pathways^114,115^. Further studies are needed to understand the roles of these factors in macrophages and HO.

## Regulation of hypoxic microenvironment by macrophages

Hypoxic microenvironment refers to a condition where the damage of the vasculature and/or the infiltration of immune cells decreases oxygen supply and/or increases oxygen consumption in cells and tissues^116^. Increasing evidence indicates that hypoxia is a critical determining factor for the appropriate microenvironment for acquired and hereditary HO^35,117^. Studies show that the primary cause of hypoxia in FOP patients is inflammation^35^. Both hypoxia and inflammation stabilize HIF-1α, and HIF-1α and HIF-1β complex upregulates the expression of BMP and VEGF, inducing MSC chondrogenic and osteogenic differentiation and angiogenesis, ultimately leading to FOP^118^ (Fig. 2) and AHO^119^. MSCs regulate hypoxia during bone development. One study^120^ has shown that the aggregation of MSCs induces local hypoxia, thereby stabilizing the transcriptional activity of HIF-1α and inducing transcription factors such as SOX9 to regulate the expression of cartilage matrix protein. The lack of HIF-1α during embryonic development may lead to joint deformities^120^. In vitro experiments have found that hypoxia can enhance the proliferation and ossification ability of MSCs^117^. Therefore, it is reasonable that inflammation-induced hypoxic microenvironment and MSCs may have a certain cascading effect in HO patients. As macrophages have been shown to be enriched in inflammation sites with severe hypoxia^121^, we propose that the immune cells that mediate the effects of hypoxia in HO patients are mainly macrophages. There is causal crosstalk between the macrophages and hypoxic microenvironment^122^. In the early phase of host response, high monocyte-macrophage extravasation, infiltration and accumulation in the inflammation site, and increased metabolic activity of infiltrating cells, together with vascular damage and edema, all contribute to reduced oxygen tension in the inflamed tissue^123^. Hypoxia, in turn, activates the inflammatory pathways to aggravate inflammation^124^. Therefore, macrophage-mediated inflammation and hypoxia signals jointly activate HIF-1α.M1 macrophages stabilize the transcription of HIF-1α in various inflammatory diseases^125,126^. For instance, the enhanced transcriptional activity of HIF-1α by macrophages has been reported in human peritoneal scar tissue^127^, suggesting that HIF-1α is expressed by different phenotypes of macrophages. In summary, it is likely that macrophages provide a suitable hypoxic microenvironment for HO and utilize multiple pathways to enhance the stability of HIF-1α to induce HO development^3^.

## Macrophages promote angiogenesis

Hypoxia and angiogenesis are inseparable processes in the body. Hypoxia response initiates the process of restoring oxygen supply found in many diseases^128,129^. At the cellular level, offsetting the lack of oxygen is achieved through actions such as angiogenesis, proliferation, and self-renewal. Hypoxia and blood vessel formation contribute to bone healing or normal bone formation^130^. For example, increased VEGF expression has been shown to promote angiogenesis and fracture healing^131^. Mice with mutant HIF-α have narrow bones and a thin cortex, which is considered to be a secondary factor of damage to the vascular system^132^. However, hypoxia and blood vessel formation are harmful processes that lead to the pathogenesis of HO. The development of HO is highly dependent on neovascularization^10,133^. Macrophages are vital factors that promote angiogenesis. Importantly, physiological angiogenesis mainly depends on tissue macrophages^134^, and numerous types of macrophages are involved in pathologic angiogenesis^135^. Under hypoxia, macrophages achieve a pro-angiogenic response directly by upregulating angiogenesis molecules (VEGF, FGF2, IL-8, VEGF type I receptors, angiogenin) or indirectly by upregulating angiogenesis regulators (SSP1, F3, MMP1)^136^. VEGF constitutes the primary vascular growth factor regulating normal and pathological angiogenesis in the body^137^. In THO and FOP mice models, macrophages have been shown to coordinate the expression of VEGF A^10^ (Fig. 2). In contrast, vascular defects have been reported in macrophage-deficient mice^134^. Mononuclear macrophages could be involved in angiogenesis and promote vascular anastomosis in HO. However, the mechanism by which macrophages of different phenotypes promote angiogenesis and induce HO remains unknown. A previous study has shown that M1 macrophages promote blood vessel generation after secreting inflammatory factors such as VEGF and TNF-α^138^, while M2 macrophages stabilize angiogenesis^138^. Therefore, VEGF A is most likely derived from M1 macrophages, and M2 macrophages assist angiogenesis in HO development. Meanwhile, the M1-M2 induced blood vessel formation is found to play an important role in the normal tissue repair process^139^. For example, after muscle injury, M1 macrophages first infiltrate the damaged site, and then M2 macrophages undergo an anti-inflammatory response and secrete cytokines (such as MMP9) to maintain fiber remodeling and vascular remodeling function^138^. In particular, IL-10 is mainly produced by macrophages, and its secretion induces blood vessel and muscle fiber formation to repair damages^140^. Depletion of M2 leads to poor injury healing^139,141^. In addition, M2 modulates EndMT in damaged and malnourished muscles. Inhibition of the M2 formation may induce EndMT in mice by activating TGF-β and promote blood vessels remodeling in the damaged muscle tissue, leading to the accumulation of collagen and the replacement of muscle by fibrotic tissue^142^. Therefore, proper expansion/maintenance of M2 in the early stage of inflammation may be needed to prevent EndMT over activity^143^ and HO. The effect of macrophages on angiogenesis could depend on when and the duration of action of different phenotypes. In conclusion, M1 and M2 macrophages are potential targets for developing an antiangiogenic therapy design for HO.

Taken together, the mechanisms that macrophages mediate HO are complex, diverse, and apparently involving the entire process of HO formation. We aim to highlight that macrophages occupy a central role by illustrating how different phenotypes macrophages mediate HO and how this will provide strategies for the prevention and treatment of HO.

## Macrophage targeting strategies in HO therapies

Macrophages are quickly recruited and activated in the inflammatory microenvironment after injury^144^. Studies have shown that different macrophage subtypes affect the inflammation outcome by secreting pro-inflammatory and/or anti-inflammatory cytokines and jointly promote the initiation and development of HO^5^. Due to the important role of macrophages in HO, the regulation of macrophage function and inflammation could effectively prevent the first episode, as well as the relapse of HO^8^.

## Immunosuppressive agents

**Fig. 3:**
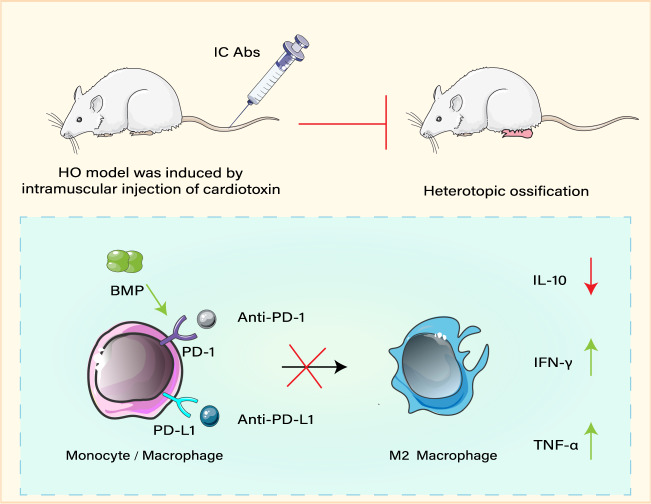
Regulation of macrophages by immune checkpoint inhibitors to block HO. Neutralizing antibodies (Abs) such as anti-PD1 and anti-PDL1 can be used to inhibit the proliferation and polarization of macrophages by targeting inhibitory ICs. These upregulate IFN-γ and TNF-α in BMP4-induced macrophages and downregulate IL-10 and decrease the population of M2, ultimately leading to the effective inhibition of HO by Abas.

Immunosuppressive agents repress immune cells, e.g., macrophages preventing an overactive immune system^145^. Currently, many clinical and experimental studies have shown that non-steroidal anti-inflammatory drugs^146^ and imatinib^147^ reduce inflammation and tissue edema, and are hence utilized as prophylaxis drugs for both acquired and inherited HO. These anti-inflammatory drugs act by inhibiting the inflammatory responses mediated by macrophages, mast cells, and other immune cells^148^, effectively fighting inflammation and suppressing the progression of HO^149^. Although these drugs are curative, they cause some adverse side effects due to their broad-spectrum immunosuppression^150^. In recent years, medications such as pexidartinib, PLX7486, and clodronate, have been used to treat cancer by suppressing the tumor-associated macrophages^151^. Interestingly, a previous study has shown that a key underlying mechanism of HO constitutes injury-induced increase of immune checkpoint proteins (ICs) expressed by macrophages, which include stimulatory ICs (CD40 and CD134) and inhibitory ICs (PD1, PD-L1, *etc*.)^51^ (Fig. 3). The use of neutralizing antibodies (Abs) against inhibitory ICs not only increases the expression of inflammatory cytokines (IFN-γ and TNF-α) in BMP4-induced macrophages, but also effectively decreases the population of CD206+/F4/80+(M2) macrophages^51^. The expression of anti-inflammatory cytokines nearly prevents or drastically limits the extent of AHO^51^, while the loss function of stimulatory ICs (CD40 and CD134) promotes HO formation^51^. It is important to note that accurate immunosuppression management suppresses the polarization of macrophages and retains mononuclear macrophages in the human body, which maintains immunity and minimizes complications^152^. Altogether, these novel immunosuppressants constitute promising drugs for the prevention and treatment of HO.

## Macrophages targeted nanomedicine

**Fig. 4:**
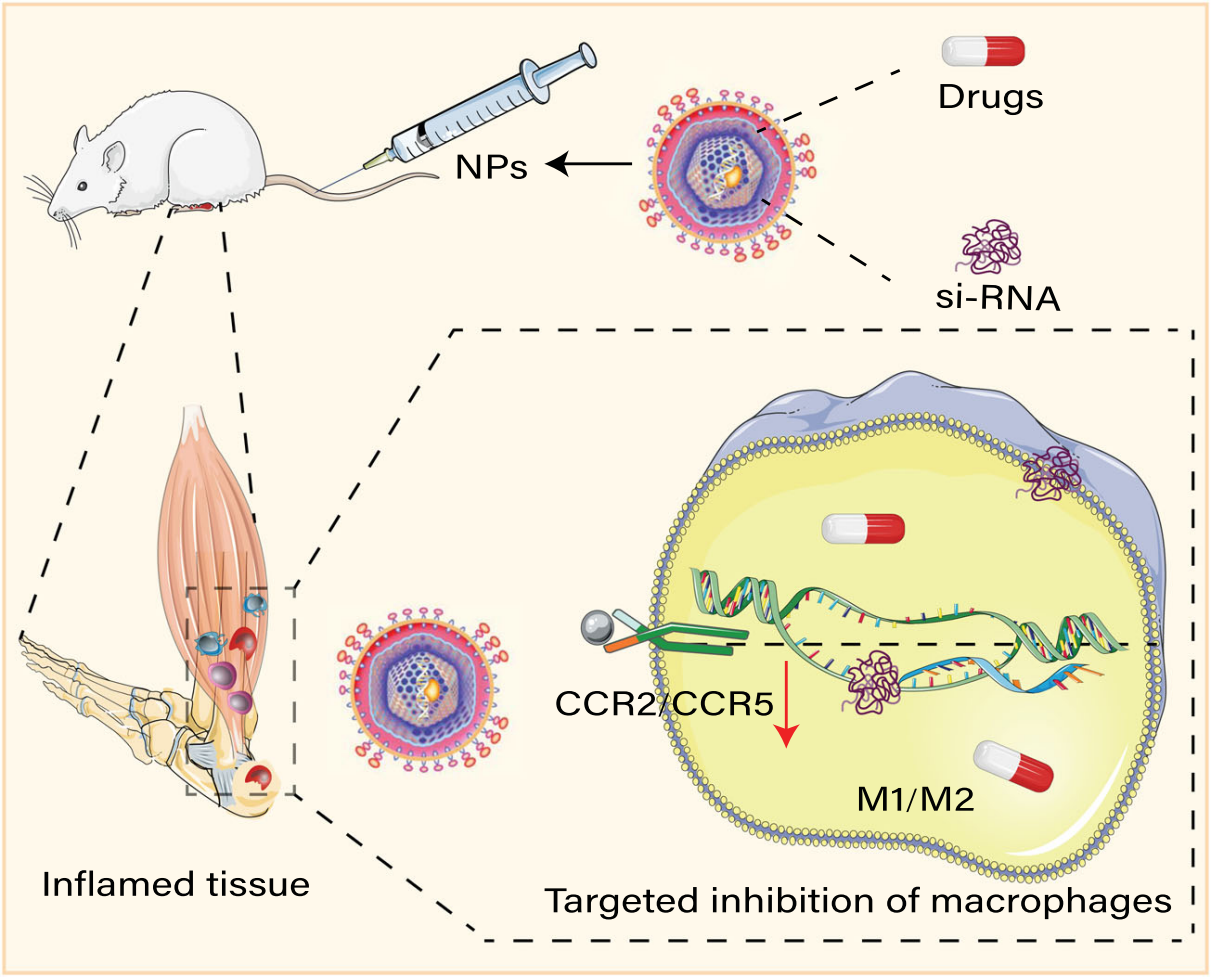
Macrophages targeted nanomedicine. Macrophages of different phenotypes in inflamed tissues can be targeted by injecting specific nanoparticles. Drug-loaded NPs, such as chlorophosphate-containing liposomes, blocks HO formation by depleting macrophages. In addition, NPs loaded with siRNA can inhibit the recruitment of macrophages and the secretion of inflammatory mediators by downregulating the expression of CCR2 or CCR5, which may be a new approach for the prevention of HO.

At present, nanomedicine mainly treats diseases with inflammation backgrounds, such as HO^153^, multiple sclerosis^154^, and others, through the development of well-designed therapies. It opens up new prospects for the treatment of inflammatory-related bone metabolism diseases. Specially designed nanoparticles (NPs) such as liposomes, solid lipid NPs, and inorganic NPs, can be used as drug carriers, targeting specific cells by identifying molecules expressed on the surface of activated macrophages^155^ or endothelial cells^153^ (Fig. 4). They thereby accurately mediate drugs to the desired cell population and regulating cell activity and functions^156^, avoiding the conventional problems of traditional medicine, such as the systemic toxicity of the drug^156^. NPs are also well-known for their enhanced permeability and retention effect. One recent study has shown that in the HO model, SPIO nanoparticles containing Smad 7 can inhibit EMT of EPCs, which is involved in PI3K/Akt signaling, depress the expression of TGF-β1 and BMP in EPC, and prevent the development of HO^153^. HO is significantly inhibited by using chlorophosphate-containing liposomes to deplete macrophages^8,157^. Nanoparticle targeting macrophages can go through two pathways-receptor-mediated endocytosis and non-specific phagocytosis^158^. M1 macrophages can be targeted by hybrid lipid–latex (LiLa) nanoparticles-bearing phagocytic signals^159^, while M2 macrophages can be targeted by mannose-decorated F127-TA hybrid nanoparticles^160^. Therefore, it may be possible to target activated macrophages through nanoparticles and administer drugs to inhibit the activation of different types of macrophages or reprogram the macrophages^155^, thereby inhibiting the development of HO. Nanomedicine not only can consume macrophages, but also can adjust the polarization bias of M1-M2 by reprogramming macrophages^161^. It is worth noting that siRNA-carrying nanoparticles also bring new possibilities for gene therapy targeting macrophages^162^. Macrophages at the injury site are recruited and differentiated from monocytes^163^. Importantly, chemokines, including CCL2 and CCL5, are vital drivers of the infiltration of monocytes/macrophages^164^, as well as of acquired and hereditary HO^29,165^. Therefore, targeting chemokines and their receptors (*e.g.*, CCL2-CCR2 and CCL5-CCR5)^164^, which effectively block the recruitment of monocytes and activation of macrophages^164^, constitutes an excellent strategy of gene therapy curing inflammatory diseases, such as HIV and multiple sclerosis^29,164^. It may be an effective way to potentially prevent and treat HO. The promising RNA-based strategies include the application of RNA silencing and antisense RNAs delivered by NPs to edit chemokine genes genetically^166^. For example, PNP/PRSsi significantly inhibits the CCR2 gene and other inflammatory genes (such as those encoding Chi3l3 and Il1rap, which are usually produced by single nuclear cell/macrophage expression), thereby reducing the recruitment of macrophages and tissue fibrosis^167^.

Other targeted therapies may also be effective in inhibiting both acquired HO and hereditary HO. Studies have shown that there is a commonality in the mechanism of AHO development and FOP-ALK2 signaling pathway. The BMP type I ALK2 receptor-specific inhibitors, such as LDN-193819 and LDN-212854, effectively inhibits more than half of AHO^82^ and FOP^63^ symptoms in rat models. At the genetic level, the use of RNA interference (RNAi) to target the expression of mutant ALK2 alleles effectively curbs the development of FOP^168^. The difference is that ACT A-ALK2 only plays a role in FOP. Treatment with anti-activin A antibody can prevent wild-type mice from forming new ectopic bones^41^. A phase II trial designed to test the efficacy of anti-activin A antibody (REGN2477) in the treatment of abnormal bone formation has recently started^169^. RARγ agonists have great potential in the prevention of AHO and FOP. RARγ agonists are likely to cause anti-chondrogenesis and prevent intramuscular and subcutaneous regions of HO by maintaining retinoid signal activity and ligand RAR activity while suppressing BMP signal transduction and phosphorylation of Smad^170–172^. In short, although different types of HO are driven by different mechanisms, the commonality in the development of acquired HO and hereditary HO in terms of inflammation, macrophages, and ALK-2 signaling pathway can be exploited to develop treatments for both types of HO.

## Conclusion

The complex pathological mechanisms underlying the occurrence and progress of HO have remained elusive for decades. Interestingly, HO does not present with typical symptoms before its occurrence, and it is irreversible once the ectopic bone is formed. Studies have focused on the critical steps of HO, including the hypoxic microenvironment, MSC osteogenic differentiation, and angiogenesis. Moreover, macrophages are recruited and activated to coordinate these three processes. More importantly, numerous studies have chronicled that the common early manifestation of HO constitutes inflammation. Herein, we elucidate the molecular mechanisms of the different phenotypes of macrophages involved in HO occurrence and progression, and highlight the potential strategies for early prevention and treatment of HO by targeting macrophages.

1. After an injury, elevated numbers of macrophages are recruited to the injury site, and together with MSCs, mast cells, chondrocytes, osteoblasts, osteoclasts, and fibroblasts form an initial inflammatory environment to mediate HO formation. The recruitment and activation of macrophages during inflammation could be the driver of HO formation and progression.
2. Macrophages constitute the major inflammation mediators in the occurrence and development of HO. Inflammation is divided into two broad categories, namely, acute and chronic. Although the type of inflammation that mediates HO has remained elusive, it is inevitable that macrophages play important roles in this inflammatory disease. This is because enriched macrophages are recruited at the early stage of inflammation, and different cytokines and factors drive the macrophage M1/M2 polarization. Disturbances in the macrophage function cause an imbalance in tissue damage and repair, leading to aberrant repair. Pro-inflammatory and anti-inflammatory cytokines are involved in HO occurrence and development, which are primarily attributed to different macrophage phenotypes.
3. Different types of macrophages drive HO by regulating MSC osteogenic differentiation and chondrogenic differentiation, hypoxic microenvironment, and angiogenesis. This is achieved through regulation of TGF-β1, BMPs, Act A, OSM, SP, and NT-3. The hypoxic microenvironment created by macrophages and stabilization of HIF-1α expression induce the formation of bone and vessels.
4. Inhibition of inflammation and macrophages with immunosuppressants and macrophage-targeting nanomedicine have the potential for HO prevention and treatment.

Many studies have noticed the prominent role of macrophages in the occurrence of HO, but mechanisms of macrophages-mediated HO remain elusive. Given the high heterogeneity and plasticity of macrophages, the inhibitory effects of different phenotypes of macrophages on bone formation and osteoinhibition vary across studies. In this review, we provide insight into the molecular mechanisms by which macrophages induce HO formation. It is likely that the sum of the osteogenic effects of macrophages in HO patients exceed the osteoinhibitory effects under pathological conditions. Proliferation and activation of osteoclasts are the only processes leading to bone resorption, but the mechanisms of ectopic bone formation promoted by macrophages are diverse and complex. Therefore it is important to explore the full landscape of molecular and cellular mechanisms by which macrophages induce HO. This will yield diagnostic and new therapeutic targets for HO.

## Data Availability

The data that support the findings of this study are available from the corresponding author upon reasonable request.
